# Immunoregulatory effects of *Lactococcus lactis‐*derived extracellular vesicles in allergic asthma

**DOI:** 10.1002/clt2.12138

**Published:** 2022-03-28

**Authors:** Dong‐Hyun Lee, Han‐Ki Park, Hee‐Ra Lee, Hyeukjun Sohn, Soyoon Sim, Hyeon Ju Park, Yoo Seob Shin, Yoon‐Keun Kim, Youngwoo Choi, Hae‐Sim Park

**Affiliations:** ^1^ Department of Allergy and Clinical Immunology Ajou University School of Medicine Suwon Korea; ^2^ Department of Allergy and Clinical Immunology School of Medicine Kyungpook National University Daegu Korea; ^3^ MD Healthcare Inc. Seoul Korea

**Keywords:** asthma, extracellular vesicles, *Lactococcus lactis*, microbiota, probiotics

## Abstract

**Background:**

Probiotics have been shown to prevent various allergic diseases by producing extracellular vesicles (EVs). However, the role of EVs in allergic asthma has not yet been completely determined.

**Methods:**

Gut microbial composition, mainly genera related to probiotics, was investigated in allergic asthmatic mice. Moreover, EVs were isolated from *Lactococcus lactis* (*L. lactis*, a selected bacterium) and EV proteins were identified by peptide mass fingerprinting. EV functions in immune responses were evaluated in vivo or ex vivo. Furthermore, the levels of specific IgG antibodies (an alternative marker for EV quantification) to *L. lactis*‐EVs were measured by ELISA in the sera of 27 asthmatic patients and 26 healthy controls.

**Results:**

Allergic asthmatic mice showed a lower proportion of Lactococcus compared to healthy mice. *L. lactis* was cultured and its EVs abundantly contained pyruvate kinase. When allergic asthmatic mice were intranasally treated with EVs, airway hyperresponsiveness, eosinophil number, cytokine secretion, and mucus production were significantly decreased. Moreover, *L. lactis*‐EV treatment shifted immune responses from Th2 to Th1 by stimulating dendritic cells to produce IL‐12. In addition, significantly lower levels of serum specific IgG4 (but not IgG1) to *L. lactis*‐EVs were noted in asthmatic patients than in healthy controls. A positive correlation between the levels of EV‐specific IgG4 and FEV_1_ (%), but a negative correlation between the levels of EV‐specific IgG4 and IL‐13 were observed.

**Conclusion:**

These findings suggest that *L. lactis‐*EVs may have immune‐regulating effects on airway inflammation mediated by dendritic cell activation, providing a potential benefit for allergic asthma.

## 1 INTRODUCTION

Asthma is a chronic inflammatory disorder of the airways primarily associated with T helper type 2 (Th2) cell‐dependent immune responses, eosinophilia and IgE production.^1^, ^2^, ^3^ In asthma pathogenesis, Th2 cells produce various cytokines, including interleukin (IL)‐5, IL‐9, and IL‐13, orchestrating immune responses.^4^, ^5^ Especially, IL‐5 contributes to eosinophilia, whereas IL‐13 is involved in mucus hypersecretion.^6^ Traditionally, dendritic cells play an important role in antigen presentation and T‐cell differentiation in lymphoid organs as a consequence of allergen exposure.^7^, ^8^ They can modulate a Th1/Th2 balance by producing IL‐12, which is essential for directing the development of Th1 cells.^9^, ^10^ In addition, bacteria have been highlighted to interact with dendritic cells in order to regulate allergic airway inflammation.

Changes in the diversity and abundance of commensal bacteria can influence asthma exacerbation by contributing to inflammation and remodeling in the lungs.^11^, ^12^ In particular, probiotics (defined as live microorganisms which confer a beneficial effect on the host) have been suggested to prevent allergic responses through multiple mechanisms.^13^ They are predominantly associated with the development of regulatory T cells to produce IL‐10, which is also known as an immunosuppressive cytokine.^14^ Among them, *Lactobacillus rhamnosus* and *Bifidobacterium breve* have been shown to induce IL‐10‐producing T cells, resulting in attenuation of airway inflammation.^15^, ^16^, ^17^ Moreover, *Lactococcus lactis* has been proposed to have an immunomodulatory function in allergic asthma.^18^, ^19^ These non‐pathogenic and non‐invasive bacteria have already been used in biotechnological applications.^20^

Extracellular vesicles (EVs; membrane‐bound organelles carrying a cargo of proteins, nucleic acids, lipids, and metabolites) are released by all cell types, including eukaryotes and prokaryotes, under physiological and pathological conditions.^21^ As these novel molecules have function in intercellular communication with a relevant effect on the immune system, EVs have been suggested to be involved in several human diseases such as cancer, metabolic disorder and allergic disease.^22^, ^23^, ^24^, ^25^, ^26^ For example, *Bifidobacterium longum* has been demonstrated to suppress mast cell activation in food allergy by producing EVs.^27^ In addition, EVs derived from *Lactobacillus plantarum* showed a protective effect against atopic dermatitis.^28^ Although the mechanism of EVs involved in asthma pathogenesis is not clear, these EVs could be a possible therapeutic agent for asthma treatment.

This study aimed to investigate (1) the relative abundance of probiotics in healthy and allergic asthmatic mice, (2) the function of EVs derived from *L. lactis* against airway hyperresponsiveness and inflammation in mice and (3) the levels of serum specific IgG1 or IgG4 to EVs associated with clinical parameters in asthmatic patients compared to healthy subjects.

## 2 METHODS

### 2.1 Establishment of allergic asthma mouse model

Animal studies were approved by the Institutional Animal Care and Use Committee of Ajou University (IACUC‐2016‐0022). All animal experiments were performed in accordance with the Guide for the Care and Use of Laboratory Animals published by Animal and Plant Quarantine Agency, Ministry of Agriculture, Food and Rural Affairs, Republic of Korea. To induce allergic asthma, each female 6‐week‐old BALB/c mouse (Jackson Laboratory, Bar Harbor, ME, USA) was intraperitoneally treated with 75 μg ovalbumin (OVA; Sigma‐Aldrich, St Louis, MO, USA) and 2 mg aluminum hydroxide (alum; Thermo Fisher Scientific, Waltham, CA, USA) in 100 μL phosphate‐buffered saline (PBS) for sensitization on day 0 and 14. Then, each OVA‐sensitized mouse was intranasally treated with 50 μg OVA in 20 μL PBS for challenges on days 28, 29, 35, 36, and 37. To evaluate airway resistance to inhaled methacholine (Sigma‐Aldrich), the flexiVent System (SCIREQ, Montreal, Canada) was used. To measure immune cell numbers in bronchoalveolar lavage fluid (BALF), Diff‐quick staining (Dade Behring, Dudingen, Switzerland) was conducted. To quantify cytokine concentrations in BALF, IFN‐γ, IL‐5, and IL‐13 were investigated using the Quantikine ELISA kit (R&D Systems, Minneapolis, MN, USA). To perform lung histological analysis, lung tissue samples were prepared a paraffin block, cut into 4‐μm‐thick sections, stained with Periodic Acid‐Schiff Kit (Sigma‐Aldrich), and observed using ImageJ (National Institutes of Health, Bethesda, MD, USA).

### 2.2 Metagenomic analysis of gut microbial diversity and composition

Bacterial DNA from fecal contents of healthy or allergic asthmatic mice was extracted by using a PowerMax Soil DNA Isolation Kit (MO BIO Laboratories Inc, San Diego, CA, USA). For the amplification of the V3 and V4 regions in the samples, primer sequences used were as follows: 319F: 5′ CCTACGGGNGGCWGCAG 3′, 806R: 5′ GACTACHVGGGTATCTAATCC 3′. To analyze the final product, paired‐end sequencing was performed using the MiSeq platform (Illumina, San Diego, CA, USA). The paired‐end data for each sample were assembled into a single sequence using FLASH (v1.2.11). The resulting sequence was passed into CD‐HIT‐out (an operational taxonomic unit analysis program based on CD‐HIT‐EST) to remove sequences of low quality, ambiguous sequences, and chimera sequences. Then, the representative sequencing of each operational taxonomic unit was performed by BLASTN (v.2.4.0) on the reference DB (NCBI 16S Microbial), and the taxonomic assignment was performed with the organism information of the subject having the highest similarity. To predict the function of clusters in each sample, Phylogenetic Investigation of Communities by Reconstruction of Unobserved States was performed. Then, the results were analyzed by the Closed‐reference operational taxonomic unit picking method of QIIME (v1.9) were used, and Greengenes DB was used as a reference.

### 2.3 Bacterial culture and EV isolation

*L. lactis* (Korean Culture Center of Microorganisms, Seoul, Korea) was cultured in Ediable MRS P medium (MD Healthcare Inc., Seoul, South Korea) under anaerobic conditions until the optical density reached 1.5 each at 600 nm. For EV isolation, bacterial culture media were centrifuged at 10,000 *g* for 20 minutes, and the supernatant was filtered through a 0.45‐μm vacuum filter. The filtrate was enriched using QuixStand (GE Healthcare, Little Chalfont, UK) and subsequently filtered through a 0.22‐μm bottle‐top filter (Sigma‐Aldrich). The filtrate was pelleted by ultracentrifugation in a 45–Ti rotor (Beckman Coulter, Fullerton, CA, USA) at 150,000 *g* for 2 hours at 4°C. The final pellets were resuspended in PBS and stored at −80°C. To observe EV shape, JEM1011 microscope (JEOL, Akishima, Japan) was used. In addition, EV size was measured using a Zetasizer Nano S (Malvern Instruments, Malvern, UK). EV protein patterns were analyzed by sodium dodecyl sulfate‐polyacrylamide gel electrophoresis.

### 2.4 Peptide mass fingerprinting

All chemicals used in this study, including 4‐sulfophenyl isothiocyanate, α‐cyano‐4‐hydroxycinnamicacid, sodium bicarbonate, and ammonium bicarbonate, were purchased from Sigma‐Aldrich. For protein identification by peptide mass fingerprinting, protein spots were excised, digested with trypsin (Promega, Madison, WI), mixed with α‐cyano‐4‐hydroxycinnamic acid in 50% acetonitrile/0.1% trifluoroacetic acid, and subjected to MALDI‐TOF analysis (Microflex LRF 20, Bruker Daltonics). Spectra were collected from 300 shots per spectrum over *m/z* range 700–4000 and calibrated by two points internal calibration using Trypsin auto‐digestion peaks (*m/z* 842.5099, 2211.1046). Peak list was generated using Flex Analysis 3.0. Threshold used for peak‐picking was as follows: 500 for minimum resolution of monoisotopic mass and 6 for S/N. The search program MASCOT, developed by The Matrixscience (http://www.matrixscience.com), was used for protein identification by peptide mass fingerprinting. The following parameters were used for the database search: trypsin as the cleaving enzyme, a maximum of one missed cleavage, iodoacetamide (Cys) as a complete modification, oxidation (Met) as a partial modification, monoisotopic masses, and a mass tolerance of ±0.2 Da.

### 2.5 EV treatment, adoptive cell transfer, and IL‐12 neutralization

For EV treatment, each OVA‐sensitized mouse was intranasally treated with 10 μg EVs (total protein concentration) and 50 μg OVA in 20 μL PBS at the same time of OVA challenges on day 28, 29, 35, 36, and 37. To compare the function of EV treatment and a conventional therapy in allergic asthma, each mouse was intraperitoneally treated with 10 μg dexamethasone (Dex; Sigma‐Aldrich) in 100 μL PBS. For adoptive cell transfer, spleen CD4^+^ T cells from wild‐type mice treated with *L. lactis*‐EVs were isolated using magnetic cell separation (Miltenyi Biotec Inc, Auburn, CA, USA). Then, they (1 × 10^5^ or 1 × 10^6^ cells in 100 μL PBS) were adoptively transferred to recipient allergic asthmatic mice the day before OVA challenges by intravenous injection. For IL‐12 neutralization, each OVA‐sensitized mouse was intraperitoneally injected with 50 μg anti‐IL‐12 antibody (R&D Systems) in 100 μL PBS during intranasal OVA challenges on days 28, 29, 35, 36, and 37. To investigate T‐cell population, flow cytometric analysis was performed using FACSAria III (BD Biosciences, San Diego, CA, USA). The antibodies used were as follows: CD4 (BD Biosciences), INF‐γ (eBioscience, San Diego, CA, USA), and IL‐13 (eBioscience). Graphs were produced by FlowJo software (Tree Star, Ashland, OR, USA). To evaluate expression of transcription factors, Western blot analysis was conducted using the antibodies as follows: T‐bet (Thermo Fisher Scientific), GATA‐3 (Abcam, Cambridge, UK), phospho‐STAT6 (Cell Signaling Technology, Danvers, MA, USA), STAT6 (Cell Signaling Technology), and actin (Santa Cruz, Dallas, TX, USA).

### 2.6 Study subjects and clinical parameters

Human studies were approved by the Institutional Review Board of Ajou University Hospital (AJIRB‐GEN‐SMP‐13‐108). All patients provided written informed consent at the time of recruitment. Additionally, experimental procedures involved in the study were conformed to the ethical consideration of the Declaration of Helsinki. The present study recruited healthy control subjects (HC; *n* = 26) and asthmatic patients (*n* = 27). The diagnosis of asthma was confirmed by allergy specialists based on clinical histories such as recurrent cough, shortness of breath, recurrent wheeze, chest tightness, and evidence of airway obstruction that was reversible after short‐acting bronchodilator inhalation according to forced expiratory volume in 1 s percentage. Asthmatic patients had used inhaled corticosteroids with/without long‐acting beta_2_ agonist, but any one had been treated with systemic corticosteroids during the experiments. Atopy status was defined as at least one positive result on skin prick tests using common inhalant allergens including house dust mites and tree/weed pollens (Bencard, Bradford, UK). Serum total IgE levels were measured by the ImmunoCAP system (Thermo Fisher Scientific). Peripheral total eosinophil counts were conducted using a hematology analyzer (Beckman Coulter, Fullerton, CA, USA).

### 2.7 Blood collection and cell isolation

Blood from patients with asthma was collected into vacutainer tubes containing acid citrate dextrose solution (BD Biosciences) by percutaneous venous catheter sampling. Blood was layered on Lymphoprep solution (Axis‐Shield, Oslo, Norway), followed by centrifugation at 800× *g* at 20°C for 25 minutes. The layer containing peripheral blood mononuclear cells was separated and red blood cells were eliminated by hypotonic lysis. Dendritic cells were analyzed and isolated using flow cytometry. The antibodies used were as follows: lineage, HLA‐DR, CD1c, and CD303 (BioLegend, San Diego, CA, USA).

### 2.8 Peripheral dendritic cell stimulation

Isolated dendritic cells were treated with 5 μg/mL human recombinant R848 (R&D Systems) or 10 μg/mL EVs for 24 h. To modify EVs, they were heated for 10 min or treated with proteinase K (10 μg/mL; Sigma‐Aldrich) for 30 min at 37°C. Moreover, dendritic cells were treated with 10 μg/mL EVs with or without SB203580 (a selective inhibitor of p38 MAPK; Cayman Chemical Company, Ann Arbor, MI, USA) in a dose‐dependent manner for 24 h. In the cell culture supernatant, concentrations of IL‐12p70 were measured using the Quantikine ELISA kits (R&D Systems), according to the manufacturer's recommendations. To evaluate the p38 pathway in the cells, the antibodies used were as follows: phospho‐p38 (Cell Signaling Technology) and p38 (Cell Signaling Technology).

### 2.9 Serum cytokine and EV‐specific antibody measurement

The levels of IL‐13 in the serum of the study subjects were measured using the Quantikine ELISA kits (R&D Systems). To evaluate the levels of EV‐specific antibodies, a 96‐well plate (Thermo Fisher Scientific) was coated with 100 ng/mL EVs derived from *L. lactis* for 12 h at 4°C. EV‐coated wells were washed twice using PBS and blocked with 1% bovine serum albumin (Sigma‐Aldrich) for 1 h at room temperature. After subsequent washing, human serum samples were incubated for 2 h. Then peroxidase‐conjugated anti‐human IgG, IgG1, and IgG4 antibodies (Sigma‐Aldrich) were incubated for 1 h. Finally, the reaction was induced by adding 3,3′,5,5′‐tetramethylbenzidine solution (BD Biosciences) and stopped by using a stop solution. The intensity was measured using a microplate reader (BioTek, Santa Clara, CA, USA) at 450 nm.

### 2.10 Statistical analysis

All statistical analyses were performed using IBM SPSS software, version 26.0 (IBM Corp., Armonk, NY, USA). Differences between two groups were analyzed by Student's *t* test. Correlations are presented as Spearman correlation coefficient *r*. *p* values of < 0.05 were considered statistically significant. GraphPad Prism 8.0 software (GraphPad Inc., San Diego, CA, USA) was used to create graphs.

## 3 RESULTS

### 3.1 Gut microbiota and functional‐gene profiles in allergic asthmatic mice

**FIGURE 1:**
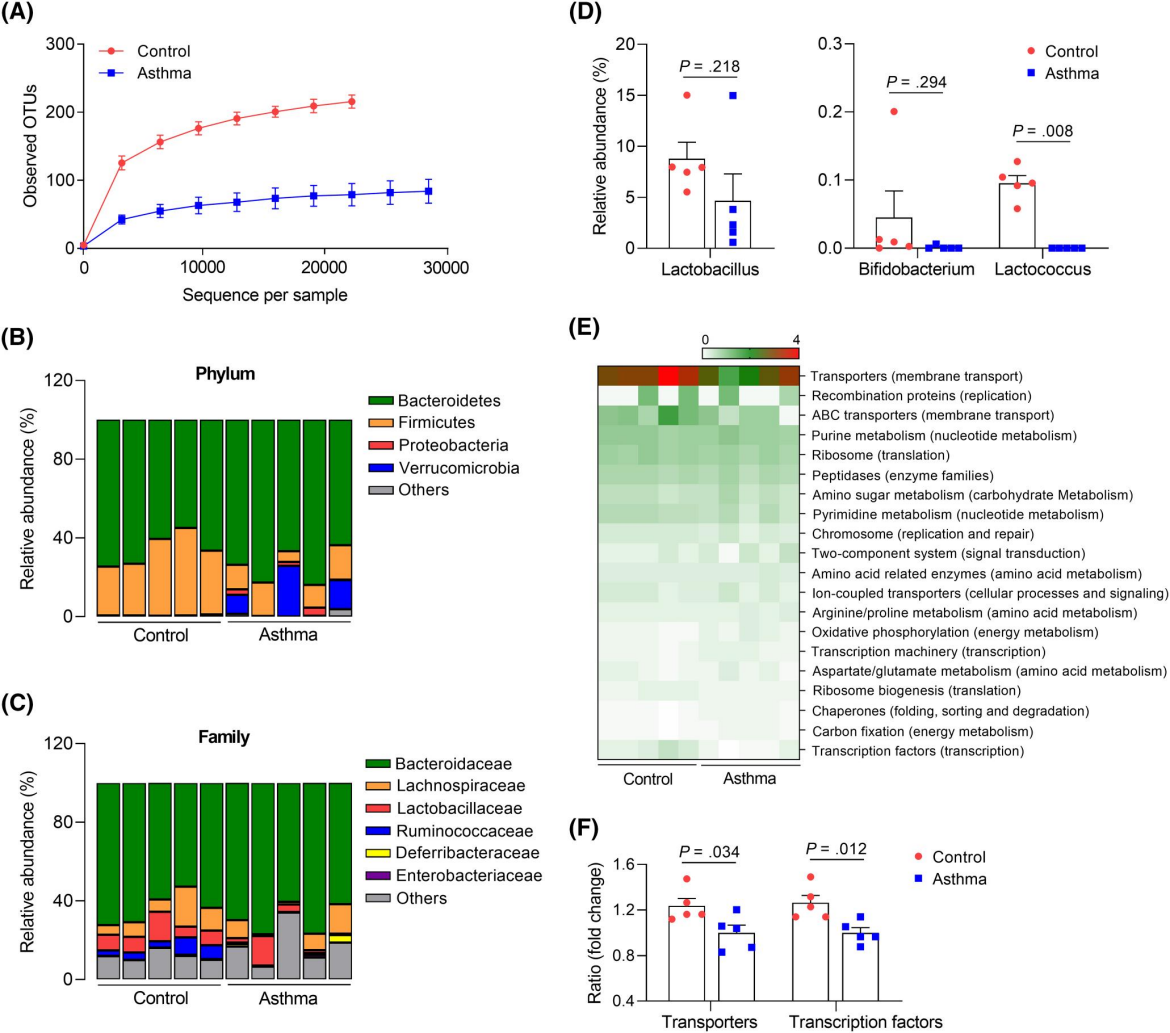
Alterations in the gut microbiota in allergic asthma *in vivo*. (A) Operational taxonomic units (OTUs). Data are presented as mean ± SD, *n* = 5. Investigation of microbial communities from the feces at the (B) phylum, (C) family, and (D) genus levels. (E) Heatmap plot of functional‐gene profiles. (F) The relative abundance of genes related to transporters and transcriptional factors. Data are presented as box plots, *n* = 5. *p* values were obtained by Student's *t* test

In allergic asthmatic mice, microbial diversity in fecal contents was markedly reduced (Figure 1A). Especially, higher proportions of Bacteroidetes, but lower proportions of Firmicutes, were shown at the phylum level (Figure 1B). Moreover, relative abundance of Bacteroidaceae increased, whereas those of Lactobacillaceae and Ruminococcaceae decreased at the family level (Figure 1C). Among genera related to probiotics, the prevalence of Lactococcus was significantly lower in asthmatic mice than in healthy mice. Although *Lactobacillus* and Bifidobacterium also tended to decrease, a statistical significance was not noted (Figure 1D). Here, we further investigated functional‐gene profiles and found that multiple genes were involved in cellular, environmental information, and genetic information processing as well as metabolism (Figure 1E). In particular, gene expression associated with transporters and transcription factors was lower in allergic asthmatic mice (Figure 1F).

### 3.2 Isolation and characterization of EVs derived from probiotics

**FIGURE 2:**
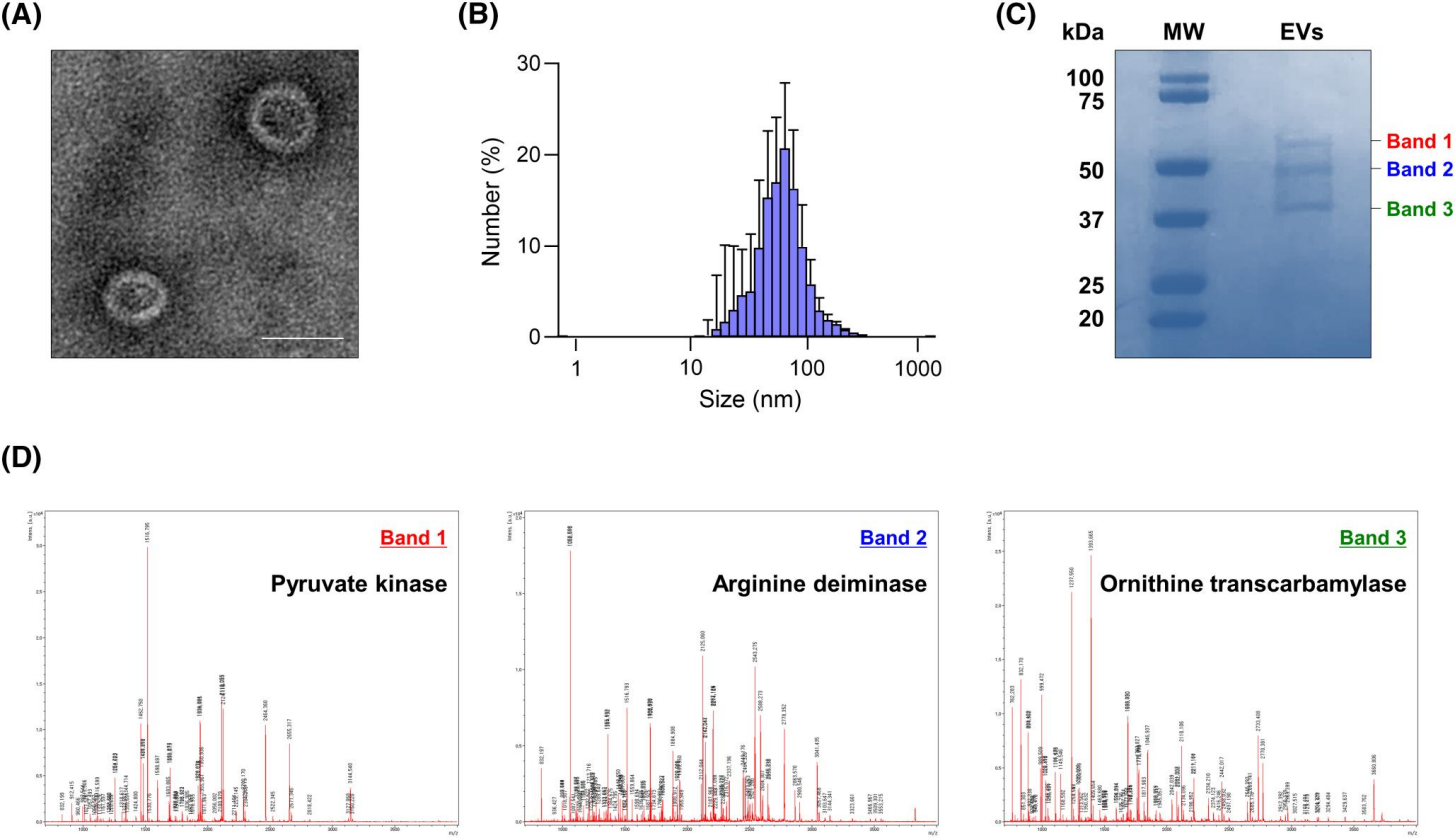
Characterization of EVs derived from probiotics. (A) EV image observed using transmission electron microscopy. Scale bar, 50 nm. (B) EV size measured using a dynamic light‐scattering analyzer. Data are represented as mean ± SD, *n* = 5. (C) Protein components in the EVs analyzed by sodium dodecyl‐sulfate polyacrylamide gel electrophoresis (D) Identification of specific proteins by peptide mass fingerprinting

The present study cultured *L. lactis* and purified their EVs. Isolated EVs showed a spherical lipid bilayer with a diameter of 60–100 nm (Figure 2A,B). Moreover, EVs were composed of various proteins (Figure 2C). Here, we further performed peptide mass fingerprinting to identify specific proteins in the EVs. As a result, pyruvate kinase as well as arginine deiminase and ornithine transcarbamylase were abundantly found in the EVs (Figure 2D).

### 3.3 Effect of *L*. *lactis*‐EVs on airway resistance and immune responses in mice

**FIGURE 3:**
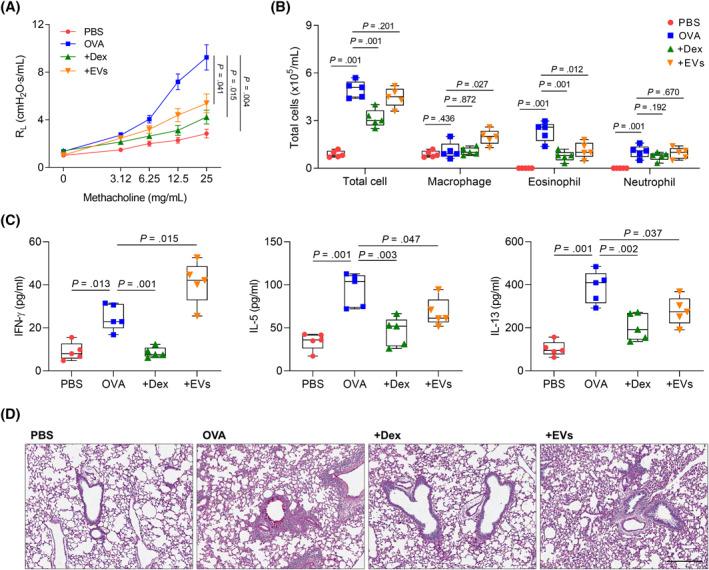
Effect of EVs derived from *L. lactis* in ovalbumin‐induced asthmatic mice. (A) Airway hyperresponsiveness. (B) Differential cell count. (C) The levels of cytokines in bronchoalveolar lavage fluid. Data are presented as box plots, *n* = 5. *p* values were obtained by one‐way ANOVA with Bonferroni's *post hoc* test. (D) Lung histology. Scale bar, 50 μm. OVA, ovalbumin; Dex, dexamethasone

The safety of EV treatment in vivo was confirmed by evaluating mouse survival rates and body weight changes (Figure S1A,B). When allergic asthmatic mice were treated with EVs or Dex, airway hyperresponsiveness, eosinophil counts, and mucus production decreased significantly. In addition, both EVs and Dex could decrease the levels of IL‐5 and IL‐13, while EVs increased IFN‐γ in the BALF of allergic asthmatic mice (Figure 3A–D). To clarify the significance of Th1/Th2 balance, adoptive T‐cell transfer was performed (Figure S1A). Proportion of IFN‐γ‐producing CD4^+^ T cells from mice (treated with the EVs) were confirmed by flow cytometric analysis (Figure S1B). By receiving spleen T cells from mice (treated with the EVs), airway hyperresponsiveness and Th2 cytokine production were significantly suppressed in allergic asthmatic mice (Figure S1C,D).

### 3.4 IL‐12‐mediated immune modulation by EV treatment in vivo or ex vivo

**FIGURE 4:**
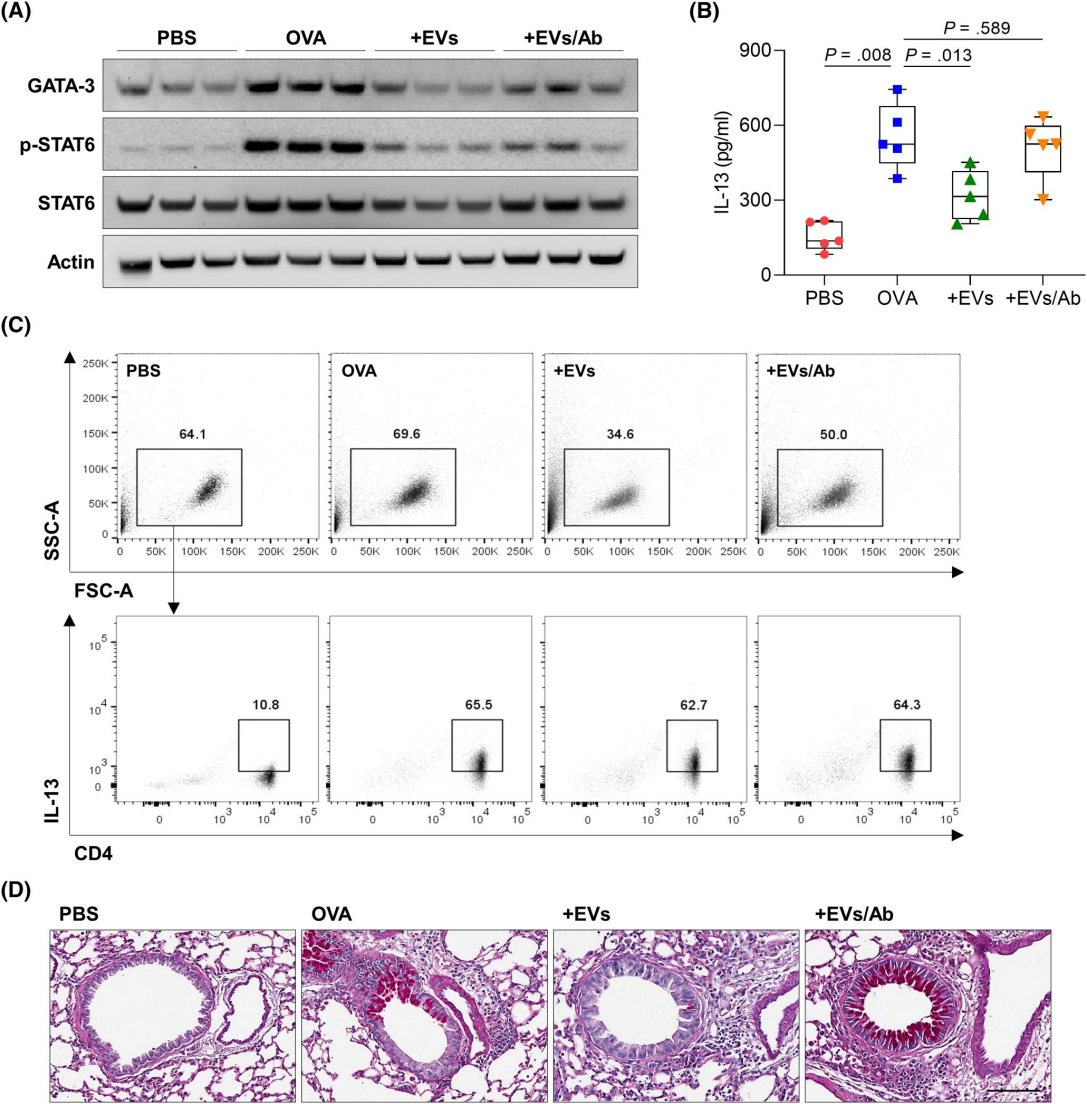
Effect of anti‐IL‐12 antibody against EV treatment in asthmatic mice. (A) Western blot analysis of GATA‐3, phospho‐STAT6, and STAT6 in the lung tissues. (B) Concentration of IL‐13 in bronchoalveolar lavage fluid. Data are presented as box plots, *n* = 5. *p* values were obtained by one‐way ANOVA with Bonferroni's *post hoc* test. (C) Flow cytometric analysis of IL‐13‐producing CD4^+^ T cells in the spleen. (D) Lung histology. Scale bar, 100 μm

**FIGURE 5:**
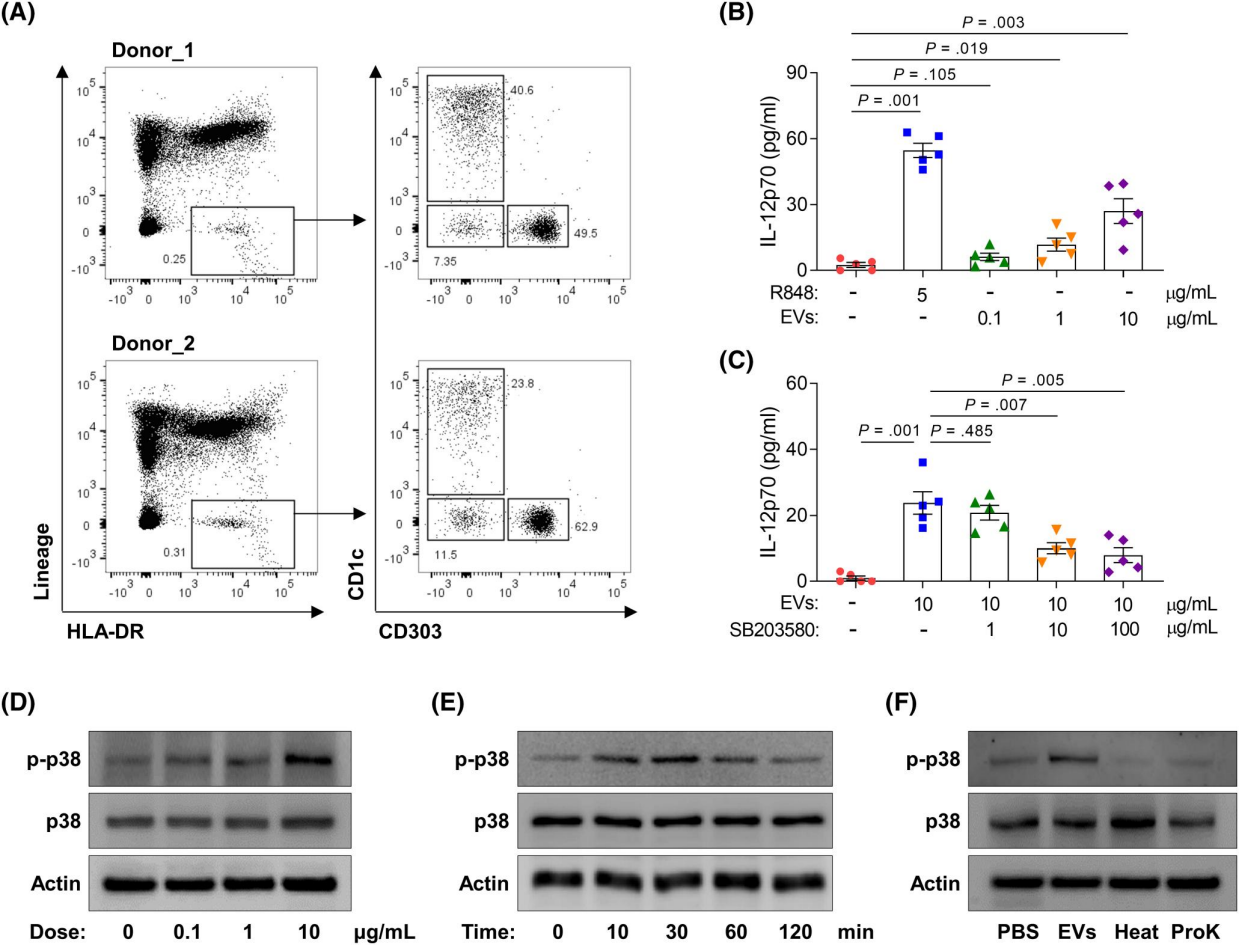
Activation of peripheral dendritic cells from healthy control subjects. (A) Identification of peripheral dendritic cell subtypes. (B) The levels of IL‐12p70 released from dendritic cells treated with multiple agents. (C) Effect of SB203580 on dendritic cell activation. Data are presented as box plots, *n* = 5. *p* values were obtained by one‐way ANOVA with Bonferroni's *post hoc* test. (D)–(F) Phosphorylation of p38 in cells treated with EVs derived from *L. lactis*. ProK, proteinase K

When allergic asthmatic mice were treated with the EVs, expression of GATA‐3 and phosphorylation of STAT6 were markedly decreased in the lung tissues; however, EVs could not show their efficacy in the presence of anti‐IL‐12 antibody (Figure 4A). Moreover, IL‐13 concentration in BALF, IL‐13‐producing CD4^+^ T‐cell proportion, and PAS‐positive area in the lungs were not decreased in allergic asthmatic mice, when EVs were treated under IL‐12 neutralization (Figure 4B–D). To demonstrate shifting from Th2 to Th1 immune responses mediated by IL‐12 production, human peripheral dendritic cells were isolated and analyzed by flow cytometry (Figure 5A). As a result, the EVs enhanced IL‐12p70 secretion from dendritic cells (Figure 5B), but the levels of IL‐12p70 in the supernatants were significantly reduced by SB203580 (Figure 5C). IL‐12p70 production induced by p38 pathway in dendritic cells was shown by Western blot analysis (Figure 5D,E). However, heated or ProK‐treated EVs could not increase p38 phosphorylation in the cells (Figure 5F).

### 3.5 Serum specific IgG antibodies to *L. lactis*‐EVs in humans

**FIGURE 6:**
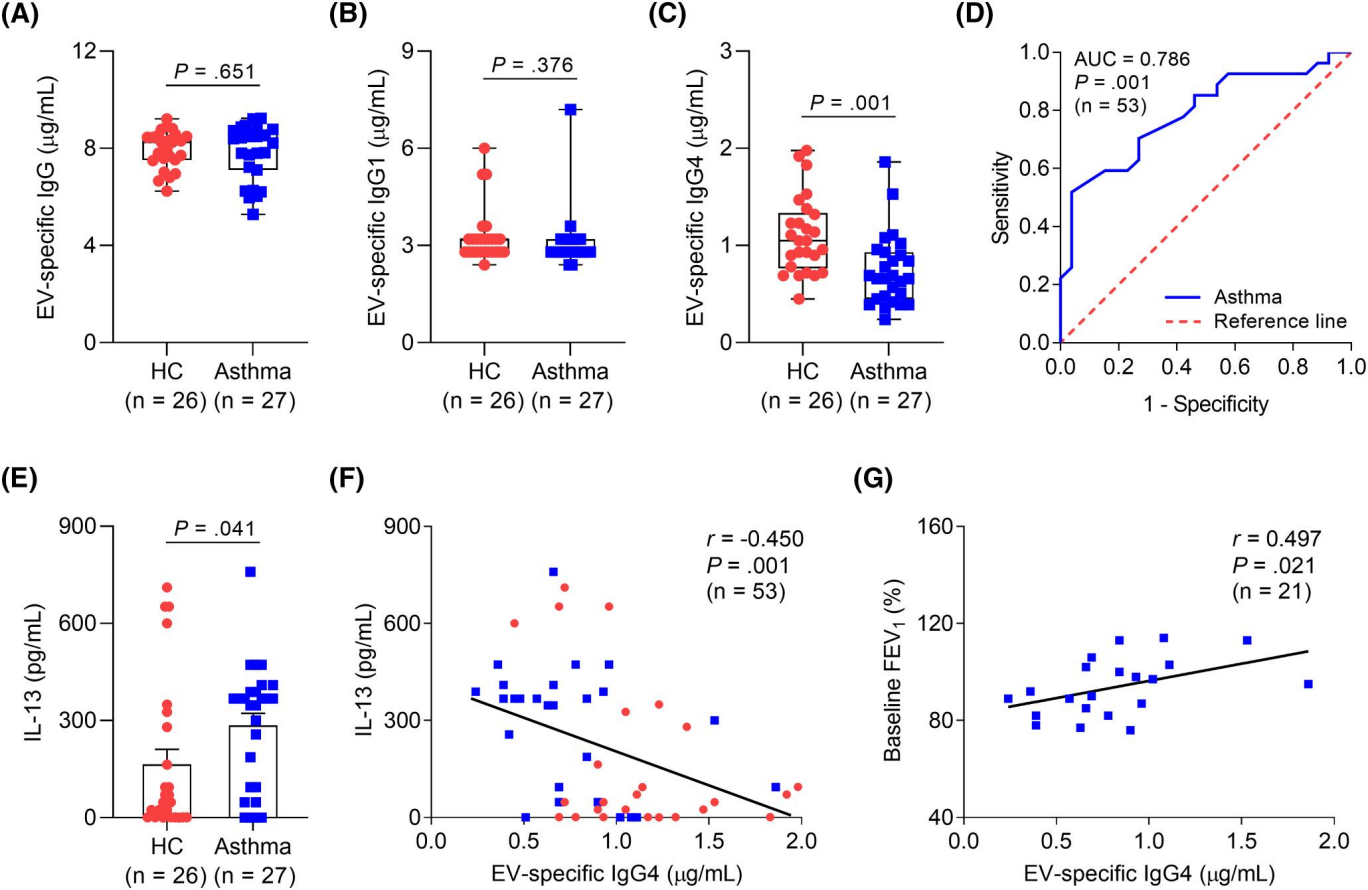
Comparison of EV‐specific IgG subclasses in the study subjects. The levels of serum specific (A) IgG, (B) IgG1, and (C) IgG4 to *L. lactis*‐EVs. (D) Receiver operating characteristic curve of EV‐specific IgG4 levels for discriminating asthmatic patients from healthy controls. (E) Concentration of IL‐13 in the serum. Data are presented as box plots. *p* values were obtained by Student's *t* test. (F) A negative correlation between the levels of EV‐specific IgG4 and IL‐13 in the study subjects. (G) A positive correlation between the levels of *L. lactis* EV‐specific IgG4 and FEV_1_ (%) in asthmatic patients. Data are presented as Spearman correlation coefficient *r* (*p* value). FEV_1_, forced exhaled volume at 1 s

To investigate the significance of *L. lactis*‐EVs in the study subjects (Table S1), the levels of EV‐specific IgG antibodies were measured in the sera of study subjects, because direct EV quantification had some limitations. The levels of serum EV‐specific IgG4 were significantly lower in asthmatic patients than in HCs, whereas those of EV‐specific IgG and IgG1 were not different between the two groups (Figure 6A–C). The receiver operating characteristic curve of the EV‐specific IgG4 levels was able to discriminate asthmatic patients from HCs (*r* = 0.786, *p* = 0.001; Figure 6D). In addition, serum IL‐13 levels were significantly higher in asthmatic patients than in HCs (Figure 6E). A negative correlation was noted between the levels of the EV‐specific IgG4 and IL‐13 (*r* = −0.450, *p* = 0.001; Figure 6F). A positive correlation was shown between the levels of the EV‐specific IgG4 and FEV_1_ (%) in asthmatic patients (*r* = 0.497, *p* = 0.021; Figure 6G).

## 4 DISCUSSION

This is the first study to demonstrate an immunomodulating effect of *L. lactis*‐EVs (rather than immune suppression) in allergic airway inflammation. These EVs could shift immune responses from Th2 to Th1 by dendritic cell activation and IL‐12p70 production, which is critical for Th1 cell differentiation. Moreover, asthmatic patients showed significantly lower levels of serum specific IgG4 to *L. lactis*‐EV in association with increased IL‐13 and decreased FEV_1_ (%). Therefore, measurement of specific IgG antibodies to EVs may replace direct quantification of EV concentrations in asthmatic patients. Taken together, these provide an insight into identification, characterization, and function of *L.lactis*‐EVs in allergic asthma.

Emerging evidence has highlighted the microbiota as a key player of the host immune system by regulating local and systemic immune responses.^29^ Especially, probiotics have been suggested to prevent allergic diseases.^30^ Probiotics are bacterial species traditionally regarded as safe; the main strains include lactic acid bacteria such as Lactobacillus and Lactococcus.^31^, ^32^ Here, we found significantly different gut microbial diversity and composition between healthy and allergic asthmatic mice. In particular, the proportion of Lactococcus species was lower in allergic asthmatic mice. Previously, *L. lactis* has been demonstrated to strongly protect against the development of childhood allergic disease in humans.^33^ Moreover, a recent paper has revealed that this bacterium prevents airway inflammation and remodeling in allergic asthma in vivo.^34^ Therefore, the present study was attempted to find the functional mechanism of *L. lactis* in the pathogenesis of allergic asthma.

This study proposed EVs derived from probiotics as key molecules having functional effects on immune regulation. Recently, the potential use of EVs as a therapeutic agent has been extensively highlighted, because these EVs could transfer biological information by an endogenous mechanism of intercellular communication.^35^ Furthermore, bacterial EVs have been implicated in human health and disease due to their roles in a wide range of biological events.^36^ To date, comparative proteomic and lipidomic analyses have identified various proteins and lipid species in EVs.^37^ Here, we showed that pyruvate kinase, arginine deiminase, and ornithine transcarbamylase were dominantly found in *L. lactis*‐EVs. Among them, pyruvate kinase has been revealed to contribute to ERK and p38 phosphorylation in cancer cells.^38^ In addition, a recent paper has demonstrated that this enzyme promotes dendritic cell activation to enhance IL‐12 expression.^39^ Although, EVs have opened a new era in studying pathophysiological processes, their diverse components and functions need to be further investigated.

In the current study, *L. lactis*‐EVs did not attenuate Th2 immune response through an IL‐10‐mediated mechanism (data not shown), although some studies have suggested that probiotics could stimulate regulatory T cells to produce an anti‐inflammatory cytokine.^40^, ^41^ Among Th2 cytokines, IL‐5 and IL‐13 have multiple functions in the development of allergic asthma. IL‐5 has been known to play a central pathogenic role in differentiation, recruitment, survival, and degranulation of eosinophils.^42^, ^43^, ^44^, ^45^ In addition, IL‐13 is a pleiotropic cytokine involved in many biological responses relevant to asthma, such as generation of eosinophil chemoattractants, maturation of mucus‐secreting goblet cells, and production of extracellular matrix proteins.^46^ Here, *L. lactis*‐EVs significantly reduced eosinophilia (IL‐5‐mediated) and mucus production (IL‐13‐mediated) in allergic asthmatic mice by enhancing Th1 immune response. Furthermore, adoptive T‐cell transfer from mice treated with *L. lactis*‐EVs to allergic asthmatic mice has revealed the significance of Th1/Th2 balance in vivo. This mechanism differs from the pathway by which corticosteroids contribute to immune suppression.

Dendritic cells play a central role in naïve T‐cell differentiation determined by the cytokine environment.^47^ IL‐12 released from dendritic cells is essential for the induction of Th1 immune response.^48^ Moreover, these cells have been shown to be stimulated by several factors including bacteria, cytokines, and simple chemicals like haptens.^49^ Here, we found that *L. lactis*‐EVs could elevate IL‐12p70 production from human peripheral dendritic cells ex vivo. Indeed, a previous study has demonstrated that *L. lactis* strains enhanced expression of IL‐12 in dendritic cells, leading to Th1 polarization.^50^ In this aspect, the development of Th1 response might control Th2‐driven allergic immune responses.^51^ Taken together, *L. lactis*‐EVs could effectively regulate allergic reactions by inducing a shift of immune responses from Th2 to Th1 rather than by inhibiting immune responses.

To date, bacterial EVs have been suggested to be a novel biomarker for allergic diseases.^26^, ^52^ However, EV‐specific antibody production or metagenomic analysis is required to detect bacterial EVs. As these processes are time consuming and expensive, we evaluated EV‐specific antibodies to show the relative abundance of bacterial EVs. As a result, significantly lower levels of specific IgG4 to *L. lactis*‐EV (but not IgG or IgG1) were observed in asthmatic patients than in HCs. Previously, asthmatic patients have been found to be more sensitized to EVs derived from bacteria, such as *Enterobacter cloacae*, *Pseudomonas aeruginosa*, and *Staphylococcus aureus,* with higher levels of EV‐specific IgG in their sera.^53^ Nevertheless, clinical implications of EV‐specific IgG subclasses have not been determined. Here, we showed a negative correlation between serum levels of EV‐specific IgG4 and IL‐13 in asthmatic patients. In addition, these EV‐specific IgG4 levels were positively correlated with FEV_1_ (%) values in asthmatic patients. These findings may provide a new approach to evaluating the role of each bacterial EV in allergic asthma.

The present study has some limitations. The one is that the relative abundance of EV composition derived from probiotics in human samples was not evaluated. The other is that direct evidence for the correlation between the concentration of bacterial EVs and the degree of bacterial exposure has not been clarified. Once these issues are clarified, the significance of bacterial EVs may be further emphasized.

In conclusion, *L. lactis*‐EVs could shift immune responses from Th2 to Th1 via stimulating dendritic cells to produce IL‐12, providing potential benefits for allergic asthma as an immunomodulator.

## CONFLICT OF INTEREST

The authors declare no competing financial interest.

## AUTHOR CONTRIBUTIONS

Dong‐Hyun Lee designed the experiments, analysed the data, and wrote the paper. Han‐Ki Park performed metagenomic data analysis and functional‐gene profiling. Hee‐Ra Lee, Hyeukjun Sohn, and Soyoon Sim performed experiments. Hyeon Ju Park cultured bacteria and isolated their extracellular vesicles. Yoo Seob Shin and Yoon‐Keun Kim helped design the experiments. Youngwoo Choi and Hae‐Sim Park provided overall supervision for the entire study.

## Supporting information

Supplementary Material
