# Supplementary material for: Immunoregulatory effects of Lactococcus lactis‐derived extracellular vesicles in allergic asthma
Source: Clin Transl Allergy. 2022 Mar 28;12(3):e12138. doi: 10.1002/clt2.12138 (PMC8967260; doi:10.1002/clt2.12138)
Supplement: Supplementary file 1 — Supplementary Material [file CLT2-12-e12138-s001.docx]

**Supporting Information**

**Immunoregulatory effects of *Lactococcus lactis*-derived extracellular vesicles in allergic asthma**

Dong-Hyun Lee,^1^ Han-Ki Park,^2^ Hee-Ra Lee,^3^ Hyeukjun Sohn,^1^ Soyoon Sim,^1^ Hyeon Ju Park,^3^ Yoo Seob Shin,^1^ Yoon-Keun Kim,^3^ Youngwoo Choi,^1*^ Hae-Sim Park^1*^

^1^Department of Allergy and Clinical Immunology, Ajou University School of Medicine, Suwon, Korea

^2^Department of Allergy and Clinical Immunology, School of Medicine, Kyungpook National University, Daegu, Korea.

^3^MD Healthcare Inc., Seoul, Korea

*These authors equally contributed to this work

**Corresponding authors:**

Youngwoo Choi

Department of Allergy and Clinical Immunology, Ajou University School of Medicine, 164 Worldcup-ro, Yeongtong-gu, Suwon, Korea.

Tel: +82-31-219-4277,

Fax: +82-31-219-4407,

Email: cyw3789@gmail.com

Hae-Sim Park MD, PhD

Department of Allergy and Clinical Immunology, Ajou University School of Medicine, 164 Worldcup-ro, Yeongtong-gu, Suwon, Korea.

Tel: +82-31-219-5196,

Fax: +82-31-219-5154,

Email: hspark@ajou.ac.kr

**Table S1. Demographic data of the study subjects**

| Variables | Healthy control subjects  (n = 26) | Asthmatic patients  (n = 27) | *P* value |
| --- | --- | --- | --- |
| Age (year) | 43.8 ± 10.3 | 44.9 ± 15.3 | .755 |
| Female sex (%) | 50.0 | 74.1 | .071 |
| Atopy (%) | 47.5 | 54.4 | .366 |
| Smoking (%) | 26.9 | 37.0 | .430 |
| Baseline FEV_1_ (%) | NA | 93.0 ± 13.1 | NA |
| PC_20_ (mg/mL) | NA | 3.3 ± 2.1 | NA |
| Total IgE (kU/L) | NA | 263.2 ± 299.1 | NA |
| TEC (cell/μL) | NA | 210.4 ± 183.2 | NA |

Values are given as n (%) for categorical variables and as mean ± SD for continuous variables.

*P* values were applied by Pearson chi-square test for categorical variables and Student’s *t* test for continuous variables.

FEV_1_, forced exhaled volume at 1 second; PC_20_, concentration of methacholine to induce a 20% decline in FEV_1_; IgE, immunoglobulin E; TEC, total eosinophil count; NA, not applicable.


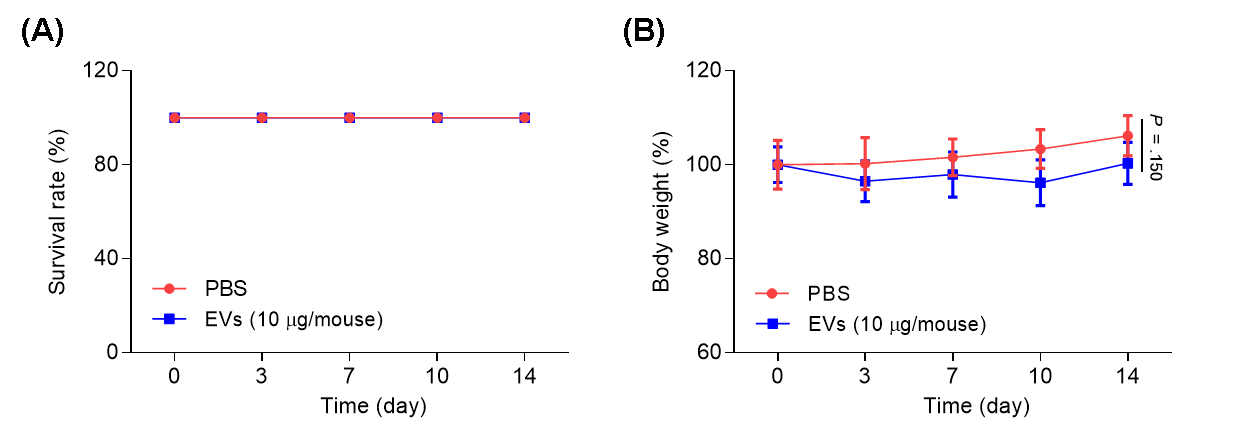


**FIGURE S1. The safety of EVs derived *Lactococcus lactis* in mice. (A)** Survival rates of mice treated with the EVs. **(B)** Changes in body weight. Data are presented as means ± SD, n = 5. *P* values were obtained by Student's *t* test. EVs, extracellular vesicles.

**
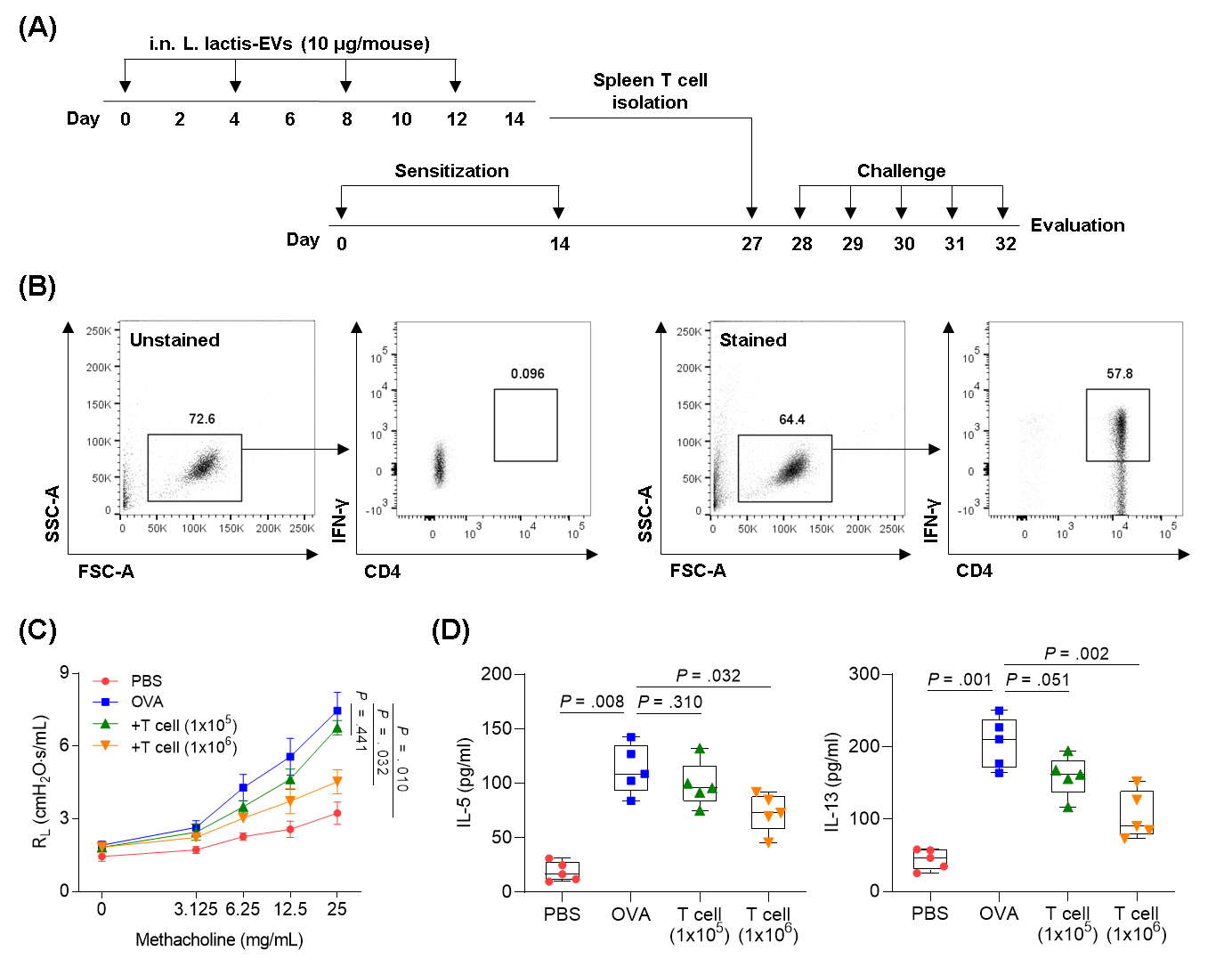
**

**FIGURE S2. Adoptive transfer of T cells from EV-treated mice to allergic asthmatic mice. (A)** Experimental protocol. **(B)** Analysis of spleen T cells using flow cytometry. **(C)** Airway hyperresponsiveness. **(D)** Levels of IL-5 and IL-13 in bronchoalveolar lavage fluid. Data are presented as box plots and dots, n = 5. *P* values were obtained using Student's *t* test.
